# Validity and prognostic value of a novel intraoperative assessment tool for reduction in trimalleolar fractures: the SGSC checklist approach

**DOI:** 10.1007/s00402-026-06390-5

**Published:** 2026-06-18

**Authors:** Hua Gao, Guoqiang Xu, Ji Ma, Yijun Wang, Jiatian Wang, Rubing Zhou, Zhenyu Liu, Baojun Wang

**Affiliations:** Department of Orthopaedics, Beijing Friendship Hospital, Beijing, China

**Keywords:** Trimalleolar fracture, Ankle arthroscopy, Fracture reduction, Assessment tool, SGSC checklist, Early radiographic degenerative changes

## Abstract

**Background:**

Trimalleolar fractures present substantial surgical challenges, and the quality of fracture reduction significantly influences patient outcomes. Current intraoperative assessment methods, including fluoroscopy and arthroscopy, each possess inherent limitations when used independently. This study aimed to validate the effectiveness of a novel intraoperative evaluation tool, the SGSC (Step, Gap, Syndesmosis, Congruity) “traffic light” checklist, that integrates arthroscopic and fluoroscopic assessment, and to evaluate its predictive value for clinical prognosis.

**Methods:**

This single-center retrospective cohort study enrolled 36 patients who underwent surgical treatment for trimalleolar fractures. Validation proceeded in two stages: first, we calculated inter-rater reliability (Kappa statistic) and concordance with postoperative CT as the reference standard to establish the reliability and validity of the SGSC checklist (accuracy validation); second, patients were stratified into “All-Green” and “Non-All-Green” groups based on intraoperative SGSC assessment, and we compared AOFAS scores and early radiographic degenerative changes at final follow-up (prognostic validation).

**Results:**

For accuracy validation, inter-rater agreement between the two assessors demonstrated excellent reliability (Kappa = 0.827; 95% CI: 0.644-1.000). The overall concordance between SGSC checklist assessments and postoperative CT findings was 91.67%. Regarding prognostic value, the All-Green group (*n* = 25) achieved a mean AOFAS score of 88.9 ± 3.0, significantly exceeding the 78.4 ± 4.4 observed in the Non-All-Green group (*n* = 11) (t = 8.32, *P* < 0.001). Furthermore, early radiographic degenerative changes (Kellgren-Lawrence grade ≥ 2) developed in only 8% (2/25) of the All-Green group compared with 45.5% (5/11) in the Non-All-Green group (*P* = 0.012).

**Conclusions:**

The SGSC checklist represents a valid and reliable intraoperative assessment instrument. Achieving “All-Green” status effectively predicts superior short-term functional outcomes and a lower incidence of early degenerative changes. However, these preliminary findings are limited by small sample size and retrospective design. Prospective studies are needed to confirm clinical utility.

## Introduction

Trimalleolar fractures constitute complex lower extremity injuries that occur frequently among both elderly and athletic populations [1, 2]. The quality of fracture reduction has been established as a critical determinant of long-term functional outcomes [3, 4]. However, currently employed intraoperative assessment modalities possess significant limitations [2, 5–9]: plain radiography often fails to detect subtle articular step-offs and gaps, whereas standard arthroscopy provides direct visualization of articular surfaces but has limited capacity for global alignment and syndesmotic stability assessment. The absence of a standardized, comprehensive, and practical evaluation tool leaves surgeons vulnerable to the dilemma of apparently satisfactory intraoperative appearances that subsequent CT imaging reveals as inadequate reduction.

To address this gap, we developed the SGSC (Step, Gap, Syndesmosis, Congruity) “traffic light” checklist as a novel intraoperative assessment instrument. This tool systematically integrates the strengths of arthroscopy (for evaluating articular Step and Gap) with fluoroscopy (for assessing Syndesmosis and Congruity). The “traffic light” terminology operates on a three-tier grading system: each of the four parameters is classified as Green (acceptable/pass), Yellow (caution/marginal), or Red (unacceptable/requires revision), creating an intuitive decision matrix for intraoperative quality control. By converting complex multi-dimensional assessments into rapid, reliable intraoperative binary judgments (pass/revision), the checklist eliminates single-modality blind spots while maintaining diagnostic rigor, thereby enhancing the practicality of arthroscopic-assisted fixation procedures.

This study was designed to validate the clinical utility of this instrument through a rigorous two-step approach: Step 1 (Validity Validation): We compared intraoperative SGSC assessments against postoperative CT as the reference standard to determine whether the tool accurately identifies reduction quality. Step 2 (Prognostic Validation): We correlated SGSC findings with short-term AOFAS scores and complication rates to establish whether the tool predicts clinical outcomes. Through these analyses, we sought to demonstrate the preliminary value of the SGSC checklist in trimalleolar fracture reduction.

## Materials and methods

### Study design and participants

Between January 2023 and December 2024, we consecutively enrolled 36 patients who underwent operative management of trimalleolar fractures in this single-center retrospective cohort study, which received institutional ethics committee approval (No. 2025-P2-302-01).

Inclusion criteria:

(1) Confirmed diagnosis of acute closed trimalleolar fracture with concurrent use of arthroscopic-assisted reduction and C-arm fluoroscopic monitoring during surgery;
(2) Complete imaging documentation, including intraoperative records and CT scanning within one week postoperatively;
(3) Minimum follow-up duration of 12 months (fracture union achieved) with complete AOFAS score documentation.
(4) Age 18–75 years.

Exclusion criteria:: Open fractures, pathological fractures, fracture-dislocations, and patients with systemic conditions known to significantly influence bone healing and functional outcomes, including diabetes mellitus, peripheral vascular disease, and active smoking.

**Fig. 1:**
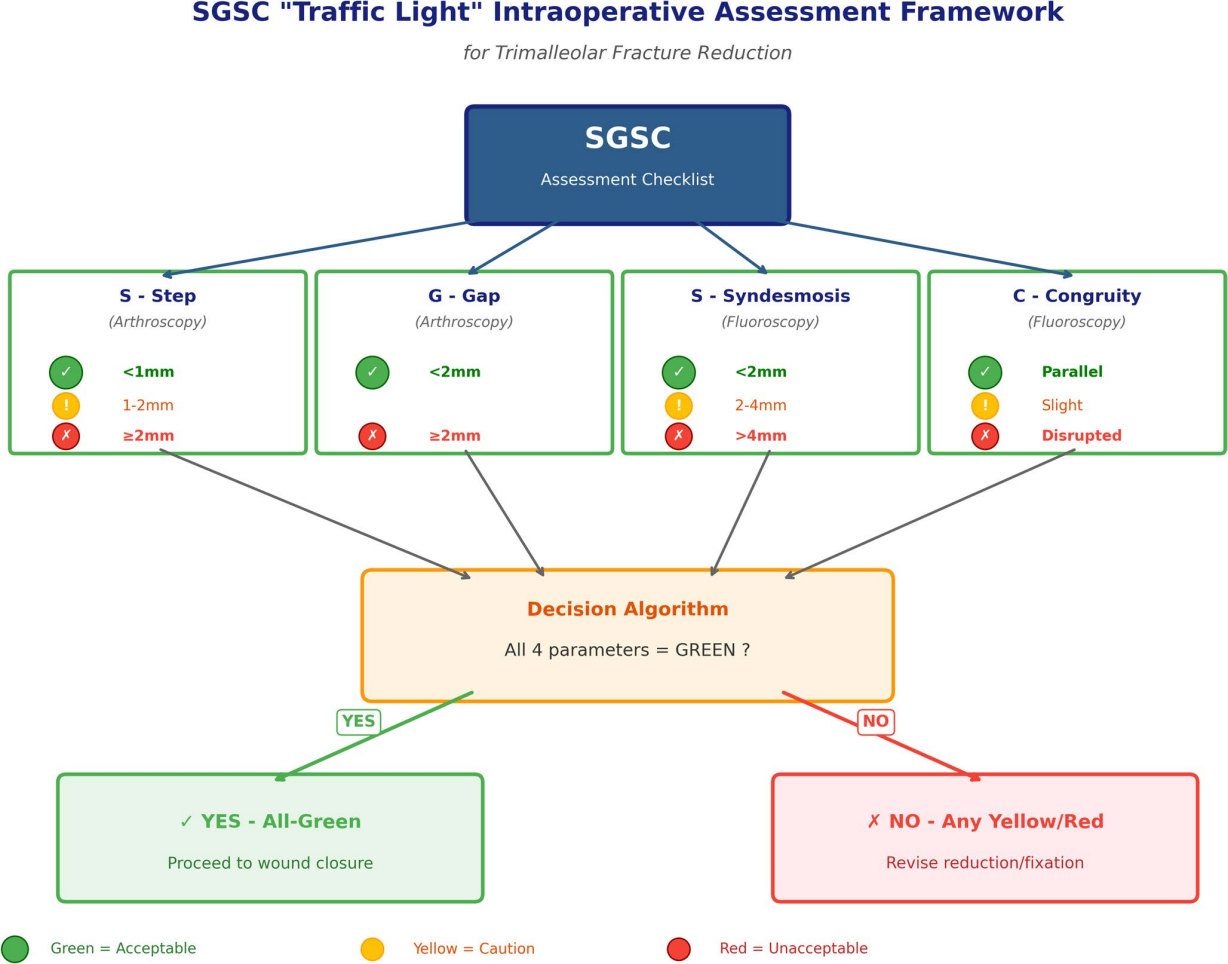
The SGSC “Traffic Light” intraoperative assessment framework for trimalleolar fracture reduction. Four parameters (Step, Gap, Syndesmosis, Congruity) are evaluated using arthroscopy and fluoroscopy. Each parameter is graded as Green (Acceptable), Yellow (Caution), or Red (Unacceptable). Only when all four parameters achieve “Green” status can the surgeon proceed to wound closure; otherwise, reduction or fixation must be revised

Surgical protocol: Following anesthesia, diagnostic and therapeutic ankle arthroscopy was performed to debride synovial tissue and remove loose bodies, with the posterior malleolar fracture site cleared of hematoma. The lateral malleolus was reduced first and temporarily stabilized with Kirschner wires. Under arthroscopic visualization, the posterior fragment was reduced using leverage and joystick techniques [10, 11] and fixed with anterior-to-posterior cannulated screws upon confirmation of satisfactory alignment. Following fluoroscopic verification of posterior malleolar reduction, the medial and lateral malleoli were definitively fixed with plates or screws according to fracture pattern [12], with syndesmotic fixation performed when indicated [13]. All reductions were intraoperatively documented using the SGSC “Traffic Light” Assessment Framework (Fig. 1). Final fluoroscopy confirmed implant positioning prior to irrigation and layered closure.

Postoperative management: All patients received standardized postoperative care. The ankle was immobilized in a below-knee plaster cast for 2 weeks, followed by a removable brace with protected weight-bearing for an additional 4 weeks. Patients with syndesmotic screws commenced full weight-bearing following screw removal at 8 weeks postoperatively [14]. All patients underwent a standardized ankle rehabilitation protocol including range-of-motion exercises and progressive strengthening. Final follow-up assessment included clinical examination, AOFAS scoring, and weight-bearing radiographs.

### Assessment methods (dual-validation framework)

Step 1: Accuracy and Reliability Validation (SGSC vs. CT).

SGSC Checklist and Quantitative Thresholds: The SGSC checklist evaluates four parameters with specific quantitative thresholds established based on anatomic and biomechanical criteria (Table 1).

**Table 1:** The SGSC (Step, Gap, Syndesmosis, Congruity) traffic-light checklist

Assessment Item	What to Evaluate	Key Tool	Green (Go/Acceptable)	Yellow (Caution/Fair)	Red (Stop/Poor)
S (Step)	Articular step-off	Arthroscopy	< 1 mm	1–2 mm	≥ 2 mm
G (Gap)	Fragment apposition	Arthroscopy	< 2 mm	–	≥ 2 mm
S (Syndesmosis)	Distal tibiofibular stability	Fluoroscopy + Hook Test	< 2 mm	2–4 mm	> 4 mm
C (Congruity)	Tibiotalar alignment	Fluoroscopy (Lateral)	Parallel arcs	Slight mismatch	Arc disrupted

Step (S): Articular step-off assessed via arthroscopy; Green < 1 mm, Yellow 1–2 mm, Red ≥ 2 mm. The < 1 mm threshold represents the minimum displacement reliably discernible under standard arthroscopic visualization.

Gap (G): Fragment apposition assessed via arthroscopy; Green < 2 mm, Red ≥ 2 mm. The < 2 mm criterion ensures anatomic apposition of fracture fragments.

Syndesmosis (S): Distal tibiofibular stability assessed via fluoroscopy and hook test; Green < 2 mm, Yellow 2–4 mm, Red > 4 mm. The threshold aligns with the definition of anatomic syndesmotic reduction.

Congruity (C): Tibiotalar alignment assessed via lateral fluoroscopy; Green = parallel arc signs, Yellow = slight mismatch, Red = arc disrupted. Parallel arc signs reflect normal tibiotalar joint morphology.

Intraoperative assessment: Two senior trauma orthopaedic surgeons of equivalent experience, blinded to postoperative outcomes, independently reviewed intraoperative arthroscopic and fluoroscopic images retrospectively. Each case was graded using the SGSC checklist across all four dimensions. The SGSC checklist was not used intraoperatively to modify surgical plans; rather, it was applied post-hoc to validate the grading system’s reliability against CT findings.

Reference standard: CT scans obtained within one week postoperatively served as the reference standard for reduction quality.

Outcome measures: Inter-rater reliability (Kappa statistic) and concordance rates between SGSC checklist assessments and CT measurements were calculated.

Step 2: Prognostic Value Validation (SGSC vs. Outcomes).

Group allocation: Based on intraoperative SGSC assessments, patients were assigned to two groups: (1) All-Green Group: All four parameters (S, G, S, C) graded “green”; (2) Non-All-Green Group: At least one parameter graded “yellow” or “red”. Given the limited sample size (*N* = 36), Yellow and Red grades were combined to ensure adequate statistical power, though individual grade distributions were preserved for future stratified analyses in larger cohorts.

Outcome measures: Groups were compared regarding American Orthopaedic Foot and Ankle Society (AOFAS) ankle-hindfoot scores at final follow-up and radiographic incidence of early degenerative changes defined as Kellgren-Lawrence grade ≥ 2 [4, 15].

### Statistical analysis

Statistical analyses were performed using SPSS version 22.0 (IBM Corp., Armonk, NY, USA). Categorical variables were analyzed using chi-squared or Fisher’s exact tests. Continuous variables with normal distribution were compared using independent samples t-tests. The Kappa coefficient assessed inter-rater reliability. Statistical significance was defined as *P* < 0.05.

Given the exploratory nature of this preliminary validation study, no formal a priori power analysis was conducted. This represents a significant methodological limitation, as the sample size of 36 patients may be insufficient to detect smaller effect sizes or to conduct robust multivariable analyses.

## Results

### Baseline characteristics

Thirty-six patients met the inclusion criteria, comprising 17 females and 19 males. Mean follow-up duration was 12.8 ± 1.8 months (Table 2). Posterior malleolar fractures were classified using the Bartoníček classification system (Type I-IV). No patients presented with fracture-dislocation, as this was an explicit exclusion criterion.

**Table 2:** Baseline characteristics of patients in each group

Group	*n*	Age	Female/Male	BMI	AO/OTA (A/B/C)	PM fracture (II/III/IV)	Syndesmosis (Y/*N*)
All-Green	25	49.0 ± 7.8	12/13	24.9 ± 3.5	6/10/9	2/12/11	17/8
Non-All-Green	11	52.5 ± 10.9	5/6	25.4 ± 4.2	2/6/3	0/7/4	4/7
**P value**	–	**0.26**	**0.89**	**0.70**	**0.38**	**0.89**	**0.073**

Age and BMI followed normal distributions, permitting independent samples t-test analysis. AO/OTA and posterior malleolar fracture classifications (Bartoníček Types II, III, IV) were ordinal categorical variables. Gender was analyzed using Pearson’s chi-squared test, while syndesmotic injury as a binary variable was assessed using Fisher’s exact test. At the alpha = 0.05 significance level, no statistically significant differences were detected between the All-Green and Non-All-Green groups regarding age, gender, BMI, AO/OTA classification, posterior malleolar fracture pattern, or syndesmotic injury (all *P* > 0.05). These findings indicate balanced baseline characteristics between groups, minimizing the likelihood of confounding in subsequent outcome comparisons, though residual confounding from unmeasured variables (e.g., smoking status, soft tissue condition) cannot be excluded.

### Accuracy validation results

We first established the measurement properties of the SGSC checklist as an assessment instrument. Reliability analysis: The two independent assessors demonstrated excellent agreement when applying the SGSC checklist (Kappa = 0.827; 95% CI: 0.645-1.000). Validity analysis: Concordance between intraoperative SGSC assessments and postoperative CT findings was 91.67%. The Kappa statistic for agreement was 0.827 (95% CI: 0.645-1.000), indicating substantial concordance. These results confirm that the SGSC checklist accurately reflects true anatomic reduction and possesses potential as an intraoperative alternative to CT assessment (Table 3).

**Table 3:** Concordance between SGSC checklist and postoperative CT findings

SGSC intraoperative score	Postoperative CT score		
	Green	Yellow	Red
Green	23	2	0
Yellow	0	8	1
Red	0	0	2

### Prognostic value validation results

Having established the accuracy of the instrument, we subsequently examined its relationship with clinical outcomes.

**Fig. 2:**
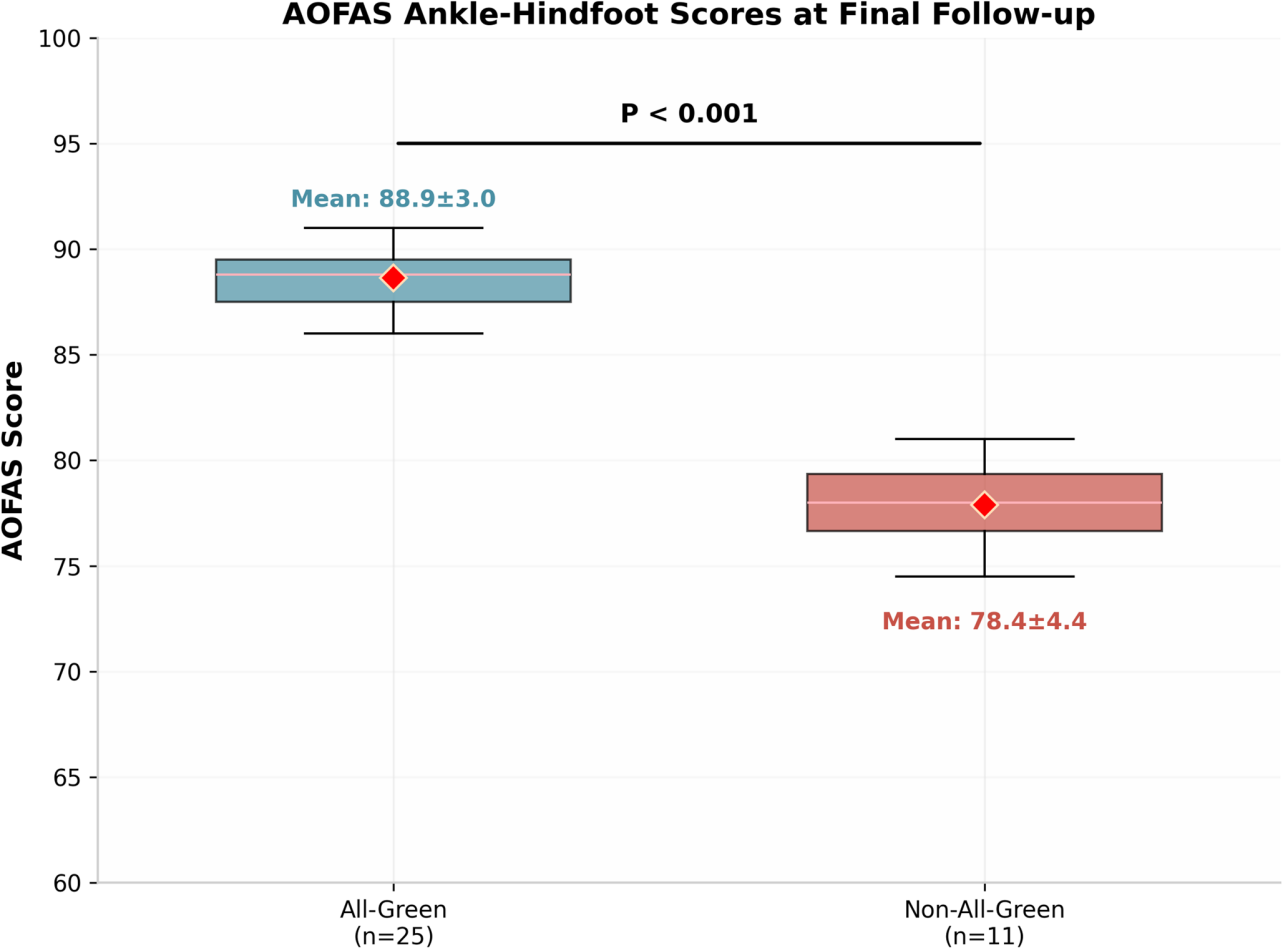
Box plot of AOFAS ankle-hindfoot scores at final follow-up between All-Green and Non-All-Green groups. The All-Green group demonstrated significantly higher scores (88.9 ± 3.0 vs. 78.4 ± 4.4, *P* < 0.001)

Functional scores (AOFAS): Postoperative functional recovery was significantly superior in the All-Green group. Mean AOFAS score was 88.9 ± 3.0 in the All-Green group (*n* = 25) compared with 78.4 ± 4.4 in the Non-All-Green group (*n* = 11) (t = 8.32, *P* < 0.001) (Fig. 2).

Early radiographic degenerative changes: Achieving “All-Green” status intraoperatively was associated with a lower incidence of early radiographic degenerative changes. Kellgren-Lawrence grade ≥ 2 changes developed in only 8% (2/25) of the All-Green group versus 45.5% (5/11) of the Non-All-Green group (*P* = 0.012). However, whether these early radiographic changes represent definitive post-traumatic osteoarthritis or merely early degenerative markers requires longer follow-up for confirmation.

## Discussion

In trimalleolar fracture management, the quality of posterior malleolar reduction directly impacts long-term functional outcomes [16, 17]. However, existing intraoperative assessment modalities, including fluoroscopy, arthroscopy, and 3D imaging, each possess significant limitations (Table 4): fluoroscopy cannot reliably identify intra-articular step-offs smaller than 2 mm; standard arthroscopy provides direct visualization of articular surfaces but has limited capacity for global alignment and syndesmotic stability assessment; and 3D imaging entails substantial costs and radiation exposure. Addressing the clinical dilemma that single-modality assessment cannot provide comprehensive evaluation, this study integrated arthroscopic direct visualization with fluoroscopic global assessment to develop the SGSC (Step, Gap, Syndesmosis, Congruity) “traffic light” checklist. Through our dual-validation design, we not only demonstrated excellent accuracy and reliability (91.67% concordance with CT, kappa = 0.827) but also established strong associations with short-term outcomes, providing a standardized solution for intraoperative reduction quality assessment.

**Table 4:** Ankle fracture reduction: intraoperative and perioperative assessment techniques review

No.	First Author	Year	Journal	Cases	Key Findings	PMID
I. Traditional/Comparative Fluoroscopy						
1	Cunningham BA [24]	2021	Foot Ankle Int	30	3D fluoroscopy changed reduction plan in 47% (14/30) of cases; final malreduction rate: 10%.	32,945,190
2	Abarka M[2]	2022	Foot Ankle Surg	52	cAPTF index > 0.161: 100% sensitivity, 97.2% specificity for predicting malreduction.	34,686,414
3	Chu X [26]	2022	Eur J Orthop Surg Traumatol	46	Contralateral template improved reduction quality; normal TFCS/SCS ratio range: 0.7–1.8.	33,890,171
II. Intraoperative 3D Imaging						
4	Franke J[27]	2012	J Bone Joint Surg Am	251	3D imaging changed surgical outcomes in 32.7%; fibular position corrected in 25.5% of patients.	22,854,991
5	Beck M [28]	2021	BMC Musculoskelet Disord	N/A	3D C-arm accurately assessed fibular position; postoperative CT can be omitted if 3D satisfactory.	33,499,849
6	Vetter SY[29]	2021	Eur J Trauma Emerg Surg	127	Validated radiological reduction criteria; anatomical reduction: Olerud score 92.44 vs. 65.47.	32,100,086
7	Spindler FT[25]	2023	Foot Ankle Int	N/A	3D imaging detected residual displacement after Suture-Button fixation; improved clinical outcomes.	36,537,750
III. Arthroscopy						
8	Wake J, Martin KD [30]	2020	J Am Acad Orthop Surg	Review	Direct visualization of distal tibiofibular joint; enables detection and management of intra-articular pathology.	32,109,919
9	Loren GJ [6]	2002	Arthroscopy	31	Articular cartilage injuries: 58% (18/31); synovial entrapment: 39% (12/31).	11,951,201
IV. Ultrasound						
10	Ghandour S [9]	2024	Arch Bone Jt Surg	4/Cadaver	Proposed ‘gap penetration sign’; specificity: 87.5%, positive predictive value: 90%.	38,577,516
11	Lu H [31]	2023	Injury	2	Standardized external rotation stress test; real-time assessment without X-ray (pilot study).	N/A
V. Direct Visualization/Palpation						
12	Pang EQ[32]	2019	J Orthop Trauma	Cadaver	Direct visualization of anterior syndesmosis more accurate than palpation for open reduction.	30,169,400
VI. CT/Weight-bearing CT						
13	Malhotra K[14]	2019	Foot Ankle Surg	26	Weight-bearing CT revealed occult instability; demonstrated fibular external rotation and posterior displacement.	30,321,955
14	Kumar V [8]	2021	J Clin Orthop Trauma	Meta/871	Meta-analysis (*n* = 871): intraoperative 3D imaging significantly improved anatomical reduction rate; reduced revision surgery.	34,040,978
VII. Comprehensive Review						
15	Hao KA [13]	2022	Curr Rev Musculoskelet Med	Review	Comprehensive review of intraoperative assessment methods; emphasizes patient-specific anatomy and comprehensive assessment strategy.	35,829,893

### Theoretical foundation and threshold determination

The SGSC checklist is founded upon the “complementary validation principle” [18–20], addressing a critical gap in current intraoperative assessment. Our systematic review (Table 4) reveals that existing modalities each possess fundamental limitations: traditional fluoroscopy lacks sensitivity for sub-millimeter articular displacement [2, 24]; intraoperative 3D imaging, while accurate, entails substantial costs and radiation exposure [23, 25]; standard arthroscopy alone provides limited assessment of syndesmotic stability and global alignment [6, 30]; and emerging techniques such as portable ultrasound and needle arthroscopy remain experimental with limited validation [9, 34, 35]. Against this fragmented landscape, the SGSC integrates arthroscopic direct visualization (for Step and Gap) with fluoroscopic global assessment (for Syndesmosis and Congruity) into a unified, standardized framework.

Each threshold was established based on anatomic and biomechanical criteria: the < 1 mm Step threshold represents the minimum displacement reliably discernible under arthroscopy, addressing the fluoroscopic blind spot identified by Cunningham et al. [24] where standard C-arm failed to detect subtle step-offs; the < 2 mm Gap criterion ensures anatomic apposition comparable to the 3D imaging standards validated by Vetter et al. [29]; the Syndesmosis threshold (< 2 mm) aligns with anatomic reduction definitions and the radiological criteria validated against cone-beam CT [28, 29]; and parallel arc signs on Congruity assessment reflect normal tibiotalar joint morphology, compensating for the arthroscopic field limitation noted by Wake and Martin [30]. This traffic-light framework converts complex multi-dimensional assessments into rapid, reliable intraoperative judgments, eliminating single-modality blind spots while maintaining diagnostic rigor (Fig. 1).

However, contemporary evidence suggests that arthroscopic evaluation of syndesmotic instability is feasible under direct visualization [31], and needle arthroscopy may further expand these capabilities [34, 35].Recent advances in needle arthroscopy (nanoscopy) offer a minimally invasive alternative with potential to enhance the SGSC concept [34, 35]. Walinga et al. [34] demonstrated that needle arthroscopy facilitates visualization of the tibiofibular syndesmosis and surrounding cartilage with minimal soft tissue disruption, while Wojtowicz et al. [35] reported successful syndesmotic repair under nanoscopic control. These developments may further enhance the applicability of arthroscopic-assisted assessment, though their integration with standardized checklist frameworks such as the SGSC requires prospective validation.

### Measurement properties and diagnostic accuracy

As a measurement instrument, the SGSC checklist demonstrates excellent psychometric properties. Inter-rater agreement was outstanding (kappa = 0.827), indicating high reliability across different observers. Concordance with postoperative CT, the reference standard for articular assessment, was 91.67%, confirming diagnostic accuracy. This performance derives directly from the complementary design: arthroscopy eliminates fluoroscopic blind spots, while fluoroscopy compensates for arthroscopic field limitations.

The SGSC integrates four assessment parameters using standard operative modalities (Table 4). Relative to the unidimensional cAPTF index [2], it expands evaluation scope, though added clinical value remains to be established. The 50% non-Green rate observed retrospectively is comparable to reported 3D imaging revision rates [23, 27, 29], but direct comparison is precluded by our study design. While wider accessibility than 3D imaging is conceivable, real-time efficacy and measurement precision require prospective validation [6, 13].

Retrospective application of SGSC criteria to the intraoperative records revealed that 18 patients (50%) had at least one parameter graded Yellow or Red, indicating that had the checklist been used intraoperatively, these cases might have warranted revision of reduction or fixation. This theoretical revision rate is comparable to that reported for intraoperative 3D imaging (52% of surgical decisions changed) [23], suggesting that the SGSC approach could provide similar diagnostic yield without additional radiation burden or equipment costs. However, because this study applied the checklist retrospectively, we cannot confirm that intraoperative use would have actually changed surgical decisions or improved outcomes. Compared with arthroscopy or fluoroscopy alone, this integrated strategy substantially enhances both sensitivity and comprehensiveness of reduction assessment.

### Prognostic validity and clinical significance

**Fig. 3:**
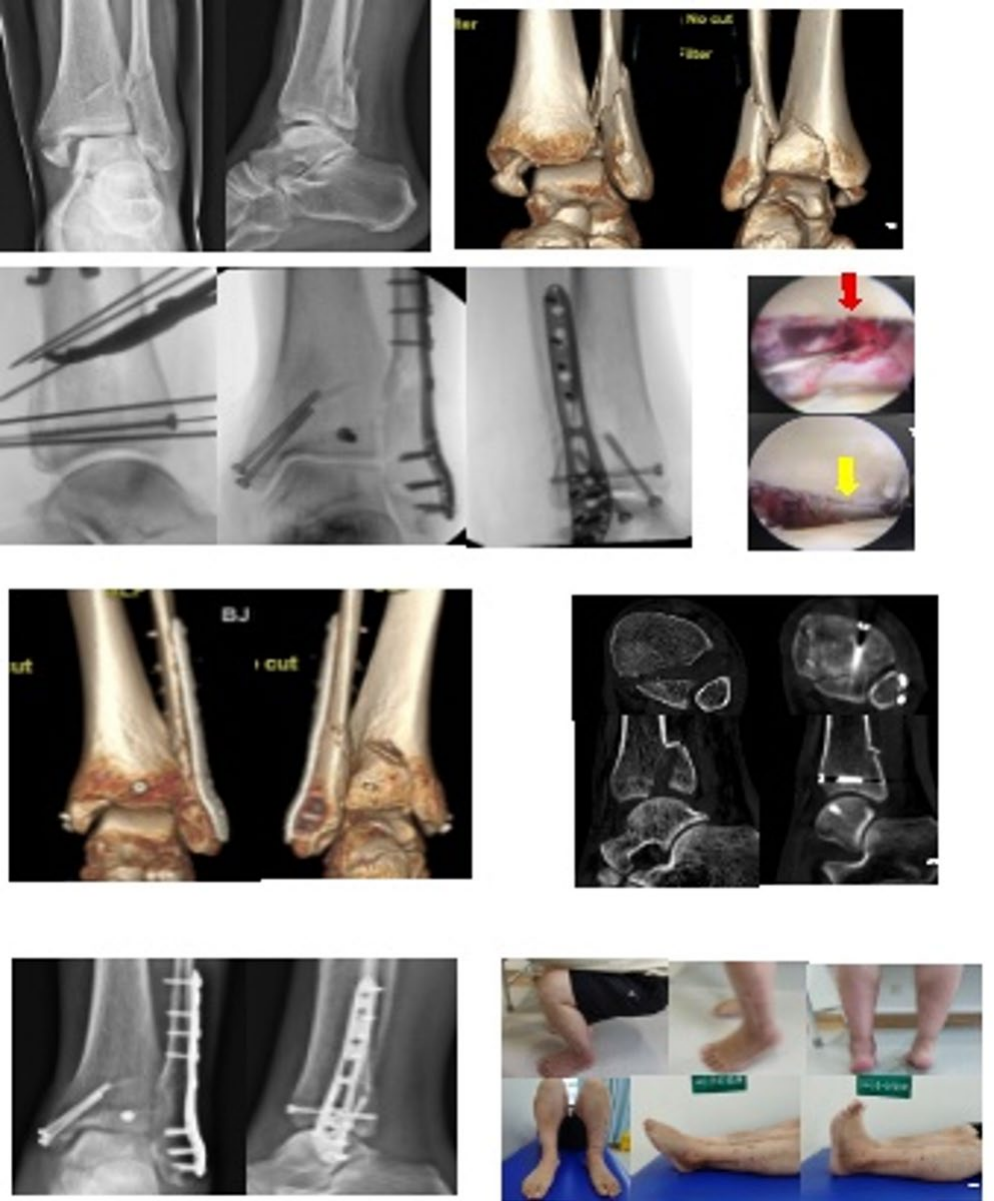
A 42-year-old male with a left trimalleolar fracture (AO/OTA 44-C2.3, Bartoníček Type IV) following a traffic accident. Preoperative imaging confirmed the diagnosis **a**,** b**. Intraoperative fluoroscopy showed satisfactory reduction with congruent joint surfaces (Congruity, green) and stable syndesmosis (Syndesmosis, green) without fixation **c**. Arthroscopy revealed pre-reduction articular step-off (red arrow) and post-reduction restoration (< 1 mm step/gap, green arrows), yielding an “All-Green” SGSC rating **d**. Postoperative CT confirmed these findings **e**, **f**. At 12 months, radiographs demonstrated solid union without arthritis (g), with an AOFAS score of 90 **h**

The SGSC checklist functions not merely as a morphologic assessment tool but as a potential prognostic indicator. Patients achieving “All-Green” status demonstrated both statistically significant and clinically meaningful advantages in short-term AOFAS scores (mean difference > 10 points, *P* < 0.001), with substantially reduced incidence of early radiographic degenerative changes (8% vs. 45.5%, *P* = 0.012) at 12-month follow-up. These findings establish a preliminary association between intraoperative reduction quality, as rigorously defined by SGSC criteria, and short-term functional outcomes [3] (Fig. 3). However, given the short follow-up duration (mean 12.8 months) and retrospective design, these results should be interpreted cautiously. The observed radiographic changes (Kellgren-Lawrence grade ≥ 2) represent early degenerative markers rather than definitive post-traumatic osteoarthritis, which typically requires longer follow-up to manifest clearly. Furthermore, the high incidence (45.5%) of these changes in the Non-All-Green group at one year may reflect either genuinely poor initial reduction or potential over-diagnosis due to the limited timeframe.

This prognostic validity, if confirmed by prospective studies, carries immediate surgical implications: the checklist provides surgeons with specific, correctable targets rather than subjective “adequate” endpoints. All “red” and “yellow” signals should be actively addressed, as residual malreduction, regardless of magnitude, correlates with inferior outcomes [24]. These results extend prior observations that anatomic reduction predicts superior patient-reported outcomes, though they require validation in larger, prospective cohorts.

### Clinical implementation and value proposition

The SGSC checklist offers distinctive advantages across multiple dimensions of surgical care. As an intraoperative quality control system, it enables real-time correction before wound closure, avoiding the passive scenario of postoperative CT findings necessitating revision surgery [8, 23, 25]. It establishes standardized terminology for surgical teams, promoting consistent understanding of reduction quality and shortening the learning curve for junior surgeons.

From a resource utilization perspective, the SGSC approach offers favorable cost-effectiveness. Unlike intraoperative 3D fluoroscopy [21, 25], it requires no additional capital investment or radiation burden; unlike arthroscopy or fluoroscopy alone, it provides comprehensive assessment without procedural redundancy. This “high-value” characteristic enhances generalizability across diverse healthcare settings [23].

### Limitations and future directions

This study has several important limitations. The single-center retrospective design, limited sample size (*N* = 36), and absence of a control group restrict statistical robustness and generalizability; findings should be considered preliminary. The 12-month follow-up is strictly short-term for assessing post-traumatic osteoarthritis, and early radiographic changes may not represent definitive degenerative disease. We excluded fracture-dislocation cases, which are known to independently worsen functional outcomes and increase PTOA risk (PMID: 40004746); however, we did not control for smoking or soft tissue condition, leaving residual confounding. The lack of a conventionally managed control group precludes superiority claims. Sub-millimeter threshold accuracy (Step < 1 mm, Gap < 2 mm) exceeds standard fluoroscopy resolution; we did not assess measurement precision or the learning curve. Finally, SGSC was applied retrospectively by independent reviewers—its utility as a real-time intraoperative decision tool remains unvalidated.

Future directions: Future investigations should prioritize multi-center prospective validation with expanded sample sizes and extended follow-up to confirm durability of functional outcomes. Cost-effectiveness analyses of SGSC implementation in ankle fracture management are warranted. Development of digital or AI-assisted checklist versions may further standardize assessment and reduce inter-rater variability. The potential integration of needle arthroscopy with the SGSC framework deserves exploration, given its minimally invasive nature and emerging evidence for syndesmotic assessment [34, 35].

## Conclusion

In summary, this preliminary validation study demonstrates that the SGSC checklist possesses excellent inter-rater reliability and substantial concordance with postoperative CT for assessing reduction quality in trimalleolar fractures. Achieving “All-Green” status was associated with superior short-term functional scores and reduced early radiographic degenerative changes.

However, these findings derive from a small, single-center retrospective cohort without a control group or a priori power analysis. The 12-month follow-up is insufficient to assess true post-traumatic osteoarthritis, and the study did not validate the checklist as a real-time intraoperative decision-making tool.

The SGSC checklist shows promise as a standardized intraoperative assessment instrument, but its routine implementation should await confirmation through multi-center prospective studies with extended follow-up, appropriate control groups, and rigorous control of clinical confounders.

## Data Availability

The datasets generated and analyzed during the current study are available from the corresponding author upon reasonable request.

## Abbreviations

SGSC: Step, Gap, Syndesmosis, Congruity
AOFAS: American Orthopaedic Foot and Ankle Society
CT: Computed Tomography
PTOA: Post-Traumatic Osteoarthritis
ORIF: Open Reduction Internal Fixation
CI: Confidence Interval
BMI: Body Mass Index
OTA: Orthopaedic Trauma Association
